# Prevalence, incidence, predictors, treatment, and control of hypertension among HIV-positive adults on antiretroviral treatment in public sector treatment programs in South Africa

**DOI:** 10.1371/journal.pone.0204020

**Published:** 2018-10-03

**Authors:** Alana T. Brennan, Lise Jamieson, Nigel J. Crowther, Matthew P. Fox, Jaya A. George, Kaitlyn M. Berry, Andrew Stokes, Mhairi Maskew, Ian Sanne, Lawrence Long, Naseem Cassim, Sydney Rosen

**Affiliations:** 1 Department of Global Health, Boston University School of Public Health, Boston, United States of America; 2 Health Economics and Epidemiology Research Office, Department of Internal Medicine, School of Clinical Medicine, Faculty of Health Sciences, University of the Witwatersrand, Johannesburg, South Africa; 3 Department of Chemical Pathology, Faculty of Health Sciences, University of the Witwatersrand, Johannesburg, South Africa; 4 National Health Laboratory Service, Johannesburg, South Africa; 5 Department of Epidemiology, Boston University School of Public Health, Boston, Massachusetts, United States of America; 6 Clinical HIV Research Unit, Department of Internal Medicine, School of Clinical Medicine, Faculty of Health Sciences, University of the Witwatersrand, Johannesburg, South Africa; 7 Right to Care, Johannesburg, South Africa; 8 Department of Molecular Medicine and Haematology, Faculty of Health Sciences, University of the Witwatersrand, Johannesburg, South Africa; University of Washington, UNITED STATES

## Abstract

**Background:**

One of the key risk factors for cardiovascular disease is hypertension. Hypertension, which leads to heart attacks and strokes, already affects one billion people worldwide, making it a global public health issue. Incidence and prevalence of the condition is on the rise in low- and middle-income countries, with the biggest increase in sub-Saharan Africa and South Africa at the forefront. We examined the prevalence, incidence, predictors, treatment, and control of hypertension among HIV-positive patients on ART in a large South African observational cohort.

**Methods:**

We conducted a prospective study of ART naïve adults initiating ART at a public sector HIV clinic in South Africa between April 2004–2017. Patients with diagnosed hypertension at ART initiation were excluded from the incidence analysis. Log-binomial regression was used to estimate predictors of hypertension at ART initiation, while competing risks regression was used to evaluate the relationship between predictors of incident hypertension, accounting for death as a competing risk.

**Results:**

Among 77,696 eligible patients, 22.0% had prevalent hypertension at ART initiation. Of the remaining patients with no hypertension at ART initiation, 8,125 incident hypertension cases were diagnosed over the period of follow-up, corresponding to an incident rate of 5.4 per 100 person-years (95% confidence interval (CI): 5.3–5.6). We found patients ≥40 years of age and patients with a body mass index (BMI) ≥25kg/m^2^ were at increased risk of both prevalent and incident hypertension. Male patients and those with pre-hypertension at ART initiation had increased hazards of hypertension over the period of follow-up. When assessing the choice of antiretroviral drug in first-line ART, patients initiated on nevirapine were at 27% increased risk of developing hypertension compared to those initiated on efavirenz, while patients who initiated on either zidovudine or stavudine had a 40% increased risk of developing hypertension compared to patients initiated on tenofovir. Patientswith poorer health status at ART initiation (i.e. WHO III/IV stage, low CD4 count, low hemoglobin levels and low BMI) had a decrease risk of prevalent hypertension. We found an inverse relationship in patients with a CD4 count <50 cells/mm^3^ at ART initiation who had a 25% increased risk of incident hypertension compared to those with a CD4 count ≥350 cells/mm^3^.

**Conclusion:**

Over 20% of patients in our cohort had hypertension at ART initiation, and 13% of those with normal blood pressure at ART initiation developed hypertension while on ART. Older patients, males, those on nevirapine, zidovudine or stavudine, and those who are overweight/obese should be targeted for frequent blood pressure monitoring and the identification of other cardiovascular risk factors to encourage lifestyle modifications. Additionally, these groups should be offered pharmaceutical therapy to help prevent myocardial infarction, heart failure, stroke, and kidney disease. Further research is needed to determine the level of access and adherence to pharmaceutical treatment for hypertension in this population. Additionally, an HIV-negative comparison population is needed to assess the association of the HIV virus itself with hypertension.

## Introduction

One of the key risk factors for cardiovascular disease is hypertension—raised blood pressure. Hypertension, which leads to heart attacks and strokes already affects one billion people worldwide, making it a global public health issue. Researchers have estimated that raised blood pressure currently kills 15 million people every year [1]. In 2017, it was estimated that 80% of the global mortality attributable to high blood pressure would occur in low and middle-income countries [1]. The importance of blood pressure as a modifiable risk factor for cardiovascular disease is well-recognized, and many effective blood pressure lowering treatments are available for the condition. Therefore, hypertension control and prevention of subsequent morbidity and mortality is achievable. However, the incidence and prevalence of the condition is on the rise in low- and middle-income countries, with the biggest increase in sub-Saharan Africa [1] and South Africa at the forefront [2].

South Africa, a middle-income country battling the largest HIV [3] and tuberculosis [4] epidemics in the world, now faces an additional challenge with an increasing burden of non-communicable chronic disease (NCD), particularly for cardiovascular disease. Incidence estimates for hypertension, one of the most common risk factors for cardiovascular disease, are lacking, but evidence indicates the prevalence of hypertension in on the rise in South Africa. In the late 1990s, the prevalence of hypertension in the general population was estimated at approximately 15% [5–7]. In 2008, Bärnighausen et al. reported a prevalence estimate of 33% among adults 15–50 years of age using Demographic Surveillance Area data from 2004 [8]. Berry et al. estimated an adult prevalence of hypertension of 35% using South African National Health and Nutrition Examination Survey data from 2011–2012 [9]. More recently, the 2016 South African Demographic and Health Survey reported 46% of women and 44% of men are hypertensive or taking antihypertensive medication [10]. The prevalence of the condition is expected to rise even faster in the coming years as obesity and sedentary lifestyles, both major risk factors for cardiovascular health, continue to increase [11].

A less-examined contributing factor to the NCD epidemic in South Africa has been the rapid expansion of HIV treatment programs [12–15]. Starting in 2004, antiretroviral therapy (ART) has been offered to HIV-positive individuals who meet a broadening set of eligibility criteria [12], which now includes all infected persons [15]. Access to ART, which has nearly reversed the country’s HIV-related loss of life-expectancy, has resulted in a steady rise in the median age of the HIV-positive population [16]. This, in turn, has increased the HIV-infected population’s risk of HIV and non-HIV related NCDs to a level comparable to that of the HIV-negative population [1]. Currently, hypertension is one of the most common risk factors for cardiovascular disease among HIV-positive individuals, with an estimated prevalence of 5% to 55% in high-income and 9% to 46% in low- and middle-income countries [17]. Studies comparing HIV positive patients to HIV negative controls have reported inconsistent results, with hypertension prevalence in HIV positive populations either comparable to [18], lower than [19,20,21], or higher than [22] their HIV negative counterparts. Some studies have found that HIV-positive individuals in sub-Saharan Africa have lower diastolic and systolic blood pressure than HIV-negative controls, regardless of ART status [19,23]. However, others have suggested an increased risk of hypertension with ART [22], and still others have shown no association between hypertension and HIV, with or without ART [18].

As the HIV-infected population ages and access to ART increases in low- and middle-income countries, the number of patients on ART developing hypertension, and subsequent CVD, will also rise, making identification and management of this condition alongside HIV increasingly important. To help fill gaps in research on this topic in low- and middle-income settings, we examined the prevalence, incidence, predictors, treatment, and control of hypertension among HIV-positive patients on ART in a large South African observational cohort.

## Methods

### Cohort description

This study used data from the Right to Care Clinical HIV cohort [24], which includes HIV positive patients from eight public-sector clinics across South Africa. The clinics began initiating patients onto ART in 2004 at the start of South Africa’s public sector treatment program. HIV care and treatment at the study clinics is provided according to national treatment guidelines [12–15]. As of April 2016, the cohort included 158,374 patients, of whom 119,397 had initiated ART. Patient data, including demographic characteristics, clinical conditions, laboratory test results, and medications (antiretroviral (ARV) and non-ARV) are entered on site by a clinician or a data entry clerk into a live data capturing system called TherapyEdge-HIV^™^. Weight, height, and systolic and diastolic blood pressure are routinely measured at medical visits.

### Eligible patients

All ART-naïve, non-pregnant, HIV-positive adult patients newly initiating standard first-line ART between April 2004 and April 2016 were eligible for the analysis. We excluded patients with missing systolic and diastolic blood pressure measurements at three months prior to one month after ART initiation. The standard first-line regimen until April 2010 was stavudine or zidovudine plus lamivudine and efavirenz; after April 2010, tenofovir or zidovudine replaced stavudine [12–15]. Mortality is routinely recorded in the patient clinical record and confirmed via linkage with the National Vital Registration system [25,26] for patients with a national identification number. For this analysis, loss to follow-up was defined as being at least three months late for the patient’s last scheduled visit to the clinic.

Use of data was approved by the Human Research Ethics Committee of the University of the Witwatersrand (M140201). Approval for analysis of de-identified data was granted by the Institutional Review Board of Boston University (H-29768).

### Outcomes

#### Primary outcomes

Our primary outcomes of interest were prevalent hypertension at ART initiation and incident hypertension on ART. Prevalent hypertension was defined as a single blood pressure (BP) measure of >140/90 mmHg three months prior to and up to one month after ART initiation, or if the patient file had hypertension noted as a condition, or if hypertension drugs had been prescribed in the same time frame. Incident hypertension was defined as a patient having normal blood pressure measurements and no evidence of previous treatment for hypertension prior to or at ART initiation and meeting any of the following criteria after ART initiation [27]:

- at least three blood pressure measurements, at least two days apart, which were >140/90 mmHg (or >130/80 mmHg in the presence of co-morbidities), OR
- a single blood pressure measure ≥180/110 mmHg, OR
- documentation in the patient file of a diagnosis of hypertension more than three months after ART initiation to ensure the patient did not have hypertension at ART initiation, OR
- documentation in the patient file of initiation of treatment for hypertension more than three months after ART initiation to ensure the patient did not have hypertension at ART initiation.

#### Secondary outcomes

We assessed three secondary outcomes:

- pre-hypertension at ART initiation, defined as a single blood pressure measurement between 120/80 mmHg and 140/90 mmHg three months prior to and up to one month after ART initiation.
- elevated blood pressure beyond the initial incident hypertension diagnosis as an indicator of whether patients’ hypertension improved over time (defined as at least three blood pressure measurements, at least two days apart, which were >140/90 mmHg (or >130/80 mmHg in the presence of co-morbidities), or a single blood pressure measure ≥180/110 mmHg).
- prevalence of hypertension-related co-morbidities, including hyperlipidemia, hypercholesterolemia, heart failure cardiomyopathy, cardiac arrest, and acute ischemic heart disease.

### Analysis

Demographic and clinical characteristics at ART initiation were summarized using descriptive statistics. For prevalent hypertension at ART initiation, we fit a log-binomial model to assess predictors (i.e. age, gender, body mass index (BMI), World Health Organization (WHO) stage of HIV-associated disease progression, CD4 count, and hemoglobin level). For incident hypertension, we fit a competing risk regression [28] model, accounting for death as a competing risk, and evaluated the relationship between incident hypertension and the following characteristics at ART initiation as potential predictors: age, gender, BMI, non-nucleoside reverse transcriptase inhibitor (NNRTI), nucleoside reverse transcriptase inhibitor (NRTI), WHO stage of HIV-associated disease progression, pre-hypertension, CD4 count, and hemoglobin level. Person time was calculated from ART initiation to either: 1) hypertension diagnosis, 2) loss to follow up (censored 3 months after last scheduled visit), 3) death, 4) transfer, or 5) close of the dataset (April 01 2016), whichever occurred first. Only patients without hypertension at ART initiation were considered for the incidence analysis. We fit linear regression models using standardized data results in standardized estimates to assess predictors of systolic and diastolic blood pressure separately. Linear models were adjusted for age, BMI, CD4 count, and hemoglobin level on a continuous scale in addition to categorical variables (i.e. gender, NRTI, NNRTI, WHO stage and pre-hypertension).

As creatinine clearance, a surrogate measure for estimated glomerular filtration rate (eGFR), was not routinely measured until tenofovir was introduced in public sector in April 2010 [13], we ran a second competing risk regression model restricted to patients who initiated after April 2010 adjusting for creatinine clearance. Creatinine clearance was calculated using the Chronic Kidney Disease Epidemiology Collaboration (CKD-EPI) equation which estimates creatinine clearance on the basis of the serum creatinine, race, age, and gender [29]. Creatinine clearance was categorized according to the U.S. National Kidney Foundation’s Kidney Disease Outcome Quality Initiative (K/DOQI) as normal (≥90ml/min), mild (60-89ml/min), moderate (30-59ml/min), and severe (<30ml/min) renal dysfunction [30]. We also adjusted for age, gender, BMI, NNRTI, NRTI, WHO stage, pre-hypertension, CD4 count, and hemoglobin level. Predictors of prevalent and incident hypertension were chosen based on previous peer reviewed studies in HIV-positive populations.

## Results and discussion

### Cohort description

A total of 80,560 patients were eligible for the analysis. Of those, 77,696 had a blood pressure measurement at ART initiation and were included in our analysis (Table 1). The cohort was predominately female (60.8%), with a median age at ART initiation of 37 years (interquartile range (IQR) 31–44)), median systolic blood pressure of 117 mmHg (106–130), median diastolic blood pressure of 76 mmHg (68–84), and median body mass index of 22.1 kg/m^2^ (19.5–25.6). Patients were initiated on a standard first-line regimen containing either stavudine (47.7%) or tenofovir (47.7%) plus lamivudine and efavirenz and had a median follow-up time of 22 months (IQR: 6–49 months).

**Table 1:** Demographic and clinical characteristics of patients on antiretroviral therapy with and without hypertension at ART initiation in Right to Care clinics in South Africa (n = 77,696).

Characteristic	Unit	Hypertension at ART initiation (N = 17,126); n (%) or median (IQR^β^)	Pre-hypertension at ART initiation (N = 15,009); n (%) or median (IQR^β^)	No hypertension at ART initiation (N = 45,561); n (%) or median (IQR^β^)
Systolic blood pressure (mm Hg)	median (IQR)	139 (125–150)	122 (113–130)	110 (101–120)
Diastolic blood pressure (mm Hg)	median (IQR)	90 (80–97)	82 (77–86)	70 (64–76)
Gender	female	10,079 (58.9%)	9,116 (60.7%)	28,024 (61.5%)
Age at ART* initiation (years)	median (IQR)	41 (34–48)	37 (32–44)	35 (30–42)
	18–24	453 (2.6%)	668 (4.5%)	3,176 (7.0%)
	25–29	1,565 (9.1%)	2,059 (13.7%)	7,843 (17.2%)
	30–39	6,200 (36.2%)	6,721 (44.8%)	20,589 (45.2%)
	40–49	5,428 (31.7%)	3,961 (26.4%)	10,235 (22.5%)
	50+	3,480 (20.3%)	1,600 (10.7%)	3,718 (8.2%)
CD4 count at ART* initiation (cells/mm^3^)	median (IQR)	144 (66–225)	127 (54–204)	115 (45–196)
	0–49	2,746 (16.0%)	2,909 (19.4%)	10,173 (22.3%)
	50–99	2,276 (13.3%)	2,101 (14.0%)	6,694 (14.7%)
	100–199	4,600 (26.9%)	4,127 (27.5%)	11,874 (26.1%)
	200–349	3,255 (19.0%)	2,386 (15.9%)	6,837 (15.0%)
	350+	1,084 (6.3%)	830 (5.5%)	2,202 (4.8%)
	missing	3,165 (18.5%)	2,656 (17.7%)	7,781 (17.1%)
WHO^€^ stage at ART* initiation	I/II	7,267 (42.4%)	5,582 (37.2%)	15,406 (33.8%)
	III/IV	4,005 (23.4%)	4,218 (28.1%)	14,804 (32.5%)
	missing	5,854 (34.2%)	5,209 (34.7%)	15,351 (33.7%)
Hemoglobin at ART* initiation (g/dL)	median (IQR)	12.2 (10.6–13.6)	11.9 (10.3–13.3)	11.4 (9.8–12.9)
	<10	2,456 (14.3%)	2,673 (17.8%)	10,480 (23.0%)
	≥10	12,091 (70.6%)	9,962 (66.4%)	27,580 (60.5%)
	missing	2,579 (15.1%)	2,374 (15.8%)	7,501 (16.5%)
Creatinine clearance (mL/min/1.73m_2_)	median (IQR)	119.1 (100.1–132.2)	123.5 (105.7–135.4)	126.2 (109.2–137.9)
	<60	391 (2.3%)	182 (1.2%)	606 (1.3%)
	60–89	978 (5.7%)	650 (4.3%)	1,602 (3.5%)
	≥90	6,957 (40.6%)	6,064 (40.4%)	17,806 (39.1%)
	missing	8,800 (51.4%)	8,113 (54.1%)	25,547 (56.1%)
Body mass Index at ART* initiation (kg/m^2^)	median (IQR)	23.7 (20.7–27.8)	22.7 (19.9–26.2)	21.4 (19.1–24.5)
	<18	940 (5.5%)	1,154 (7.7%)	5,395 (11.8%)
	18–24.9	6,765 (39.5%)	6,561 (43.7%)	21,333 (46.8%)
	25–29.9	3,106 (18.1%)	2,372 (15.8%)	5,380 (11.8%)
	30–34.9	1,379 (8.1%)	924 (6.2%)	1,634 (3.6%)
	>35	787 (4.6%)	379 (2.5%)	675 (1.5%)
	missing	4,149 (24.2%)	3,619 (24.1%)	11,144 (24.5%)
Tuberculosis at ART* initiation	yes	1,384 (8.1%)	1,575 (10.5%)	5,727 (12.6%)
Non-nucleotide reverse transcriptase Inhibitor	efavirenz	15,817 (92.4%)	13,769 (91.7%)	41,158 (90.3%)
	nevirapine	1,152 (6.7%)	1,105 (7.4%)	3,832 (8.4%)
	missing	157 (0.9%)	135 (0.9%)	571 (1.3%)
Nucleoside reverse transcriptase Inhibitor	zidovudine	852 (5.0%)	609 (4.1%)	1,616 (3.5%)
	stavudine	8,162 (47.7%)	7,570 (50.4%)	24,006 (52.7%)
	tenofovir	8,112 (47.4%)	6,830 (45.5%)	19,939 (43.8%)
Time on ART* (months)	median (IQR)	24.8 (9.2–49.7)	23.9 (9.0–49.3)	21.6 (7.4–47.5)
Vital status at end of follow-up period	died	1,480 (8.6%)	1,276 (8.5%)	5,047 (11.1%)
	loss to follow-up	3,578 (20.9%)	3,449 (23.0%)	11,471 (25.2%)
	transferred out	3,959 (23.1%)	3,609 (24.0%)	10,838 (23.8%)
	alive	8,109 (47.3%)	6,675 (44.5%)	18,205 (40.0%)

*ART = antiretroviral therapy;

^€^WHO = World Health Organization;

^β^IQR = interquartile range.

### Prevalent hypertension

More than a fifth of the cohort (n = 17,126, 22.0%) had prevalent hypertension at ART initiation. As expected, older patients (40–49 –hazard ratio (HR): 1.49; 95% confidence interval (CI):1.42–1.54 and >50–2.00; 1.91–2.11) and patients classified as overweight or obese (BMI 25–29.9 kg/m^2^ –HR: 1.39; 95% CI: 1.32–1.45; 30–34.9 kg/m^2^ –HR: 1.70; 95% CI: 1.60–1.81; and ≥35 kg/m^2^ –HR: 1.96; 95% CI: 1.83–2.10) had a higher prevalence of hypertension at ART initiation. However, individuals initiating ART with poorer health status (i.e. WHO staging III/IV, and hemoglobin <10g/dL) had lower prevalent hypertension (Table 2).

**Table 2:** Predictors of prevalent hypertension in patients on ART (n = 77,696).

Variable	Number with baseline hypertension (%)	Unadjusted Risk Ratio (95% CI^¥^)	Adjusted Risk Ratio (95% CI^¥^)
**Age**			
18–24	453 (10.5%)	0.57 (0.52–0.62)	0.60 (0.52–0.69)
25–29	1,565 (13.6%)	0.74 (0.70–0.78)	0.73 (0.68–0.79)
30–39	6,200 (18.5%)	Reference	Reference
40–49	5,428 (27.7%)	1.49 (1.45–1.54)	1.49 (1.42–1.56)
50+	3,480 (39.6%)	2.14 (2.07–2.21)	2.00 (1.91–2.11)
**Gender**			
Female	10,079 (21.3%)	Reference	Reference
Male	7,047 (23.1%)	1.08 (1.05–1.11)	1.13 (1.08–1.18)
**Body mass index (kg/m**^**2**^) at ART* **initiation**			
<18	940 (12.6%)	0.64 (0.60–0.69)	0.72 (0.67–0.78)
18–24.9	6,765 (19.5%)	Reference	Reference
25–29.9	3,106 (28.6%)	1.47 (1.41–1.52)	1.39 (1.32–1.45)
30–34.9	1,379 (35.0%)	1.79 (1.71–1.88)	1.70 (1.60–1.81)
≥35	787 (42.7%)	2.19 (2.07–2.32)	1.96 (1.83–2.10)
**WHO**^€^ **stage**			
I/II	7,267 (25.7%)	Reference	Reference
III/IV	4,005 (17.4%)	0.68 (0.65–0.70)	0.84 (0.80–0.87)
CD4 count at ART* **initiation (cells/μl)**			
0–49	2,746 (17.3%)	0.66 (0.62–0.70)	0.93 (0.85–1.01)
50–99	2,276 (20.6%)	0.78 (0.73–0.83)	0.99 (0.91–1.08)
100–199	4,600 (22.3%)	0.85 (0.80–0.90)	1.01 (0.94–1.10)
200–349	3,255 (26.1%)	0.99 (0.93–1.05)	1.08 (1.00–1.18)
350+	1,084 (26.3%)	Reference	Reference
Hemoglobin at ART* **initiation (g/dL)**			
> = 10	12,091 (24.4%)	Reference	Reference
<10	2,456 (15.7%)	0.65 (0.62–0.67)	0.79 (0.75–0.84)

*ART = antiretroviral therapy;

^€^WHO = World Health Organization;

^¥^CI = confidence interval. All variables in the adjusted model are displayed in the table.

### Incident hypertension

**Fig 1:**
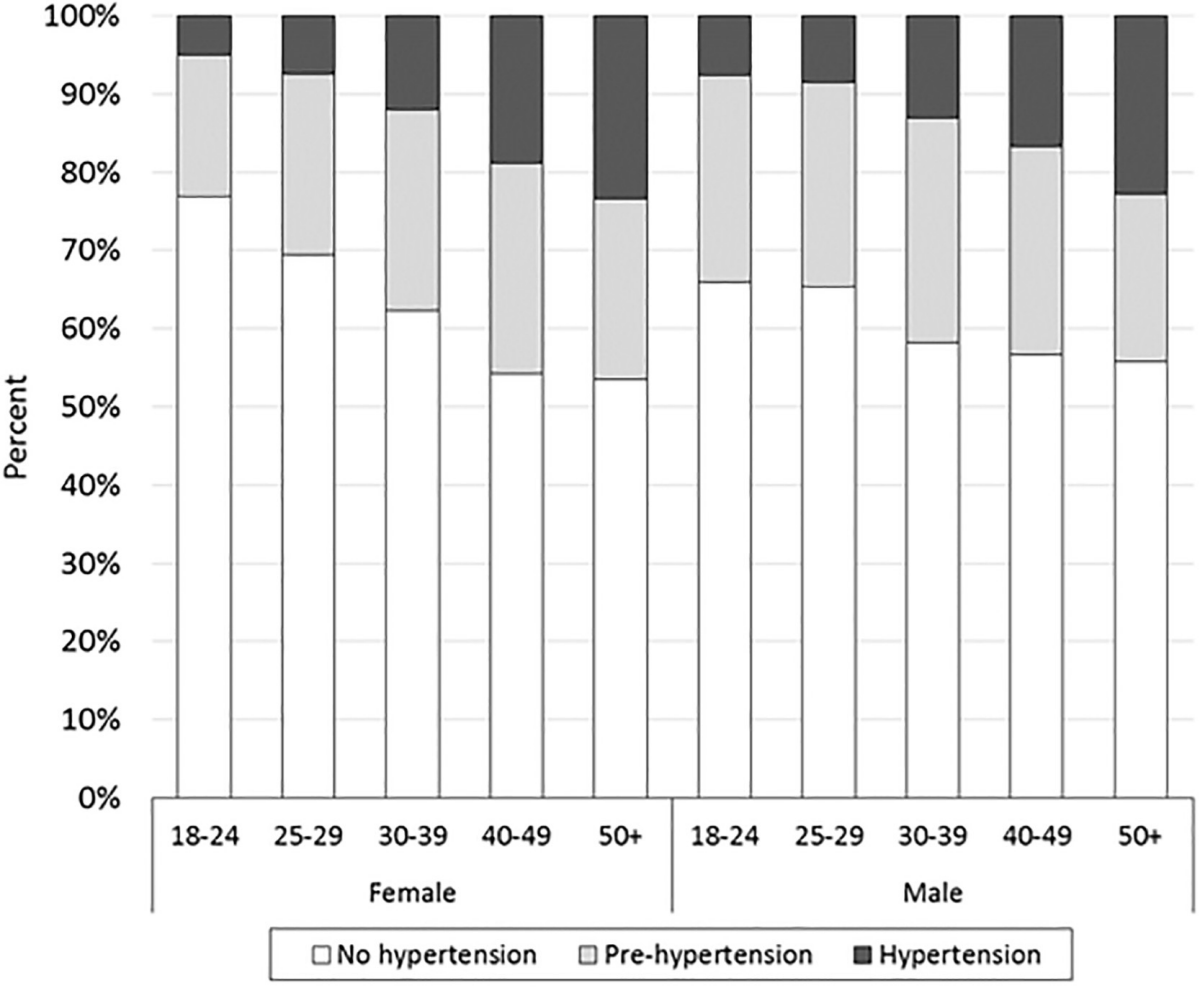
Proportion of study population with incident hypertension and pre-hypertension after ART initiation, stratified by age and gender (N = 60,570).

For our assessment of incident hypertension while on ART, we excluded patients with hypertension at ART initiation (n = 17,126), leaving a final sample of 60,570. Of these, 75.2% (n = 45,561) had normal blood pressure and 24.8% (n = 15,009) patients were pre-hypertensive. There were 8,125 (13.4%) incident hypertension cases diagnosed at a median of 13 months (IQR: 9–230) after ART initiation. The overall incidence rate for this cohort was 5.44 per 100 person years (95% CI: 5.32–5.56). Men had a higher rate of incident hypertension (6.25 per 100 person years; 95%CI: 6.04–6.46) than women (4.98 per 100 person years; 95%CI: 4.84–5.12) overall. By age, men had higher incidence of pre-hypertension and hypertension than women until the oldest two age groups (40–49 and ≥50 years), where women showed higher rates than men (Fig 1). Results from the linear regression models indicate that a change of one standard deviation in the in BMI resulted in a 0.175 (95% CI: 0.142, 0.208) standard deviation increase in the systolic blood pressure and a 0.251 (0.218, 0.285) standard deviation increase in the diastolic blood pressure (Table 3). Additionally, a change of one standard deviation in the in BMI resulted in a 0.209 (95% CI: 0.196, 0.222) standard deviation increase in diastolic blood pressure.

**Table 3:** Linear regression analysis on systolic and diastolic blood pressure showing both normal and standardrized parameter estimates of predictors (n = 60,570).

Variable		Parameter estimates		Standardized parameter estimates	
		Unadjusted Unstandardized β estimate (95% CI^¥^)	Adjusted unstandardized β estimate (95% CI^¥^)	Unadjusted standardized β estimate (95% CI^¥^)	Adjusted standardized β estimate (95% CI^¥^)
**Systolic blood pressure**					
Age (per 1 year increase)		-0.021 (-0.037, -0.007)	-0.064 (-0.088, -0.039)	-0.012 (-0.020, -0.004)	-0.034 (-0.47, -0.021)
Gender	Female	**Reference**	**Reference**	**Reference**	**Reference**
	Male	1.483 (1.210, 1.756)	2.208 (1.764, 2.652)	0.043 (0.035, 0.051)	0.065 (0.052, 0.078)
Body mass index (per kg/m^2^ increase)		.054 (0.043, 0.065)	0.216 (0.175, 0.257)	0.044 (0.035, 0.053)	0.175 (0.142, 0.208)
Pre-hypertension at ART* initiation	No	**Reference**	**Reference**	**Reference**	**Reference**
	Yes	4.291 (3.98, 4.598)	3.167 (2.684, 3.651)	0.111 (0.103, 0.119)	0.082 (0.070, 0.095)
NNRTI^α^	efavirenz	**Reference**	**Reference**	**Reference**	**Reference**
	nevirapine	-0.183 (-0.668, 0.303)	0.182 (-0.621, 0.984)	-0.003 (-0.011, 0.005)	0.003 (-0.010, 0.016)
NRTI^Ω^	tenofovir	**Reference**	**Reference**	**Reference**	**Reference**
	zidovudine	-1.361 (-2.083, -0.638)	-2.304 (-3.532, -1.076)	-0.015 (-0.024, -0.007)	-0.026 (-0.040, -0.012)
	stavudine	-2.959 (-3.230, -2.688)	-2.506 (-2.947, -2.065)	-0.089 (-0.097, -0.081)	-0.075 (-0.089, -0.062)
CD4 count (per 100 cells/μl)		0.006 (-0.002, 0.013)	-0.0002 (-0.011, 0.0107)	0.007 (-0.002, 0.016)	-0.0002 (-0.013, 0.013)
WHO^€^ stage	I/II	**Reference**	**Reference**	**Reference**	**Reference**
	III/IV	-2.914 (-3.244, -2.584)	-1.891 (-2.320, -1.463)	-0.088 (-0.097, -0.078)	-0.057 (-0.070, -0.044)
Hemoglobin (per 1 g/dL increase)		0.061 (0.044, 0.079)	0.025 (-0.0001, 0.049)	0.031 (0.022, 0.040)	0.013 (-0.0001, 0.025)
**Diastolic blood pressure**					
Age (per 1 year increase)		0.487 (0.463, 0.511)	0.447 (0.407, 0.487)	0.161 (0.153, 0.169)	0.148 (0.135, 0.161)
Gender	Female	**Reference**	**Reference**	**Reference**	**Reference**
	Male	2.265 (1.821, 2.708)	2.168 (1.444, 2.892)	0.041 (0.033, 0.049)	0.039 (0.026, 0.052)
Body mass index (per kg/m^2^ increase)		0.073 (0.055, 0.092)	0.503 (0.436, 0.571)	0.037 (0.027, 0.046)	0.251 (0.218, 0.285)
Pre-hypertension at ART* initiation	No	**Reference**	**Reference**	**Reference**	**Reference**
	Yes	14.238 (13.749, 14.726)	13.088 (12.299, 13.876)	0.227 (0.220, 0.235)	0.209 (0.196, 0.222)
NNRTI^α^	efavirenz	**Reference**	**Reference**	**Reference**	**Reference**
	nevirapine	-0.229 (-1.018, 0.560)	1.588 (0.278, 2.897)	-0.002 (-0.010, 0.006)	0.016 (0.003, 0.029)
NRTI^Ω^	tenofovir	**Reference**	**Reference**	**Reference**	**Reference**
	zidovudine	5.477 (4.303, 6.650)	6.597 (4.593, 8.601)	0.038 (0.030, 0.046)	0.046 (0.032, 0.060)
	stavudine	4.964 (4.524, 5.404)	7.752 (7.033, 8.471)	0.092 (0.084, 0.100)	0.143 (0.130, 0.157)
CD4 count (per 100 cells/μl)		0.009 (-0.021, 0.003)	0.014 (-0.004, 0.032)	0.007 (-0.002, 0.016)	0.010 (-0.003, 0.024)
WHO^€^ stage	I/II	**Reference**	**Reference**	**Reference**	**Reference**
	III/IV	-2.404 (-2.951, -1.857)	-0.870 (-1.569, -0.170)	-0.044 (-0.055, -0.034)	-0.016 (-0.029, -0.003)
Hemoglobin (per 1 g/dL increase)		0.055 (0.027, 0.083)	0.012 (-0.028, 0.052)	0.017 (0.008, 0.026)	0.004 (-0.009, 0.016)

*ART = antiretroviral therapy;

^€^WHO = World Health Organization;

^¥^CI = confidence interval;

^α^NNRTI = Non-Nucleoside Reverse Transcriptase Inhibitor;

^Ω^NRTI = Nucleoside reverse transcriptase inhibitor. All predictors are measured at ART initiation. Model adjusted for gender, body mass index, age, nucleoside reverse transcriptase inhibitor, non-nucleoside reverse transcriptase inhibitor, WHO stage, CD4 count at ART initiation, hemoglobin levels at ART initiation.

Similar to prevalent hypertension, we found that patients ≥40 years of age and those classified as overweight or obese were at increased hazards of hypertension during their time on ART (Table 4). Male patients (HR: 1.23; 95% CI: 1.14–1.32) and those with pre-hypertension (HR: 2.05; 95% CI: 1.92–2.19) at ART initiation also had increased hazards of hypertension over the period of follow-up (Table 4). Patients with a CD4 count <50 cells/mm^3^ at ART initiation had a 25% increased hazards of developing hypertension compared to those with a CD4 count ≥350 cells/mm^3^. When assessing the choice of NNRTI in first-line ART, patients initiated on nevirapine were at 27% increased hazards of developing hypertension compared to patients initiated on efavirenz (HR: 1.27; 95% CI: 1.13–1.43), while patients who initiated on either zidovudine or stavudine had a 40% increased hazards of developing hypertension compared to patients initiated on tenofovir (Table 4).

**Table 4:** Predictors of incident hypertension in patients on ART (n = 60,570).

Variable	Number of events/person years	Incidence rate/100 person years (95% CI^¥^)	Crude hazard ratio (95% CI^¥^)	Adjusted hazard ratio (95% CI^¥^)
**Gender**				
Female	4,699/83,557	5.62 (5.46–5.79)	Reference	Reference
Male	3,426/47,144	7.27 (7.03–7.51)	1.22 (1.17–1.28)	1.23 (1.14–1.32)
Age (years) at ART* **initiation**				
18–24	208/7,406	2.81 (2.44–3.22)	0.50 (0.43–0.57)	0.54 (0.44–0.67)
25–29	761/21,818	3.49 (3.24–3.74)	0.64 (0.60–0.70)	0.74 (0.66–0.82)
30–39	3,396/62,283	5.45 (5.27–5.64)	Reference	Reference
40–49	2,532/30,528	8.29 (7.97–8.62)	1.49 (1.41–1.57)	1.45 (1.35–1.57)
≥50	1,228/8,665	14.17 (13.39–14.99)	2.15 (2.01–2.30)	1.99 (1.80–2.20)
**Body mass index (kg/m**^**2**^) at ART* **initiation**				
<18	674/13,430	5.02 (4.65–5.41)	0.72 (0.66–0.78)	0.72 (0.65–0.80)
18.0–24.9	3,756/64,355	5.84 (5.65–6.03)	Reference	Reference
25.0–29.9	1,304/17,556	7.43 (7.03–7.84)	1.34 (1.25–1.43)	1.38 (1.27–1.51)
30.0–34.9	515/5,232	9.84 (9.01–10.73)	1.74 (1.58–1.91)	1.87 (1.64–2.13)
≥35	215/1,958	10.98 (9.56–12.55)	1.88 (1.63–2.16)	2.01 (1.66–2.45)
Pre-hypertension at ART* **initiation**				
No	4,805/100,828	4.77 (4.63–4.90)	Reference	Reference
Yes	3,320/29,873	11.11 (10.74–11.50)	2.32 (2.22–2.43)	2.05 (1.92–2.19)
**Non-Nucleoside Reverse Transcriptase Inhibitor**				
efavirenz	7,335/117,486	6.24 (6.10–6.39)	Reference	Reference
nevirapine	679/11,346	5.98 (5.54–6.45)	1.00 (0.93–1.09)	1.27 (1.13–1.43)
**Nucleoside Reverse Transcriptase Inhibitor**				
tenofovir	2,368/41,118	5.76 (5.53–6.00)	Reference	Reference
zidovudine	379/5,171	7.33 (6.61–8.11)	1.32 (1.18–1.48)	1.41 (1.18–1.69)
stavudine	5,378/84,412	6.37 (6.20–6.54)	1.17 (1.11–1.23)	1.42 (1.31–1.54)
CD4 count at ART* **initiation (per 100 cells/μl)**				
0–49	1,900/27,708	6.86 (6.55–7.17)	1.09 (0.96–1.24)	1.25 (1.03–1.50)
50–99	1,327/19,613	6.77 (6.41–7.14)	1.15 (1.01–1.31)	1.15 (0.95–1.39)
100–199	2,238/38,678	5.79 (5.55–6.03)	1.08 (0.95–1.22)	1.05 (0.87–1.26)
200–349	1,102/18,751	5.88 (5.54–6.23)	1.08 (0.95–1.23)	1.13 (0.93–1.37)
350+	0,279/4,945	5.64 (5.00–6.34)	Reference	Reference
**WHO**^€^ stage at ART* **initiation**				
I/II	3,175/49,997	6.35 (6.13–6.58)	Reference	Reference
III/IV	2,613/42,053	6.21 (5.98–6.46)	0.86 (0.81–0.90)	1.00 (0.93–1.07)
Hemoglobin at ART* **initiation (g/dL)**				
<10	1,690/25,104	6.73 (6.41–7.06)	0.90 (0.85–0.96)	1.06 (1.00–1.12)
≥10	5,472/87,415	6.26 (6.10–6.43)	Reference	Reference

*ART = antiretroviral therapy;

^€^WHO = World Health Organization;

^¥^CI = confidence interval. All predictors are measured at ART initiation. Model adjusted for gender, body mass index, age, nucleoside reverse transcriptase inhibitor, non-nucleoside reverse transcriptase inhibitor, WHO stage, CD4 count at ART initiation, hemoglobin levels at ART initiation. Fine and Gray’s competing risk regression used controlling for death as a competing risk for incident hypertension

When limiting the cohort to those patients who initiated ART after the South African national guidelines called for the replacement of stavudine with tenofovir in first-line ART (April 1, 2010), we found that patients with moderate/severe renal insufficiency (creatinine clearance <60 ml/min) had a 70% increase in the hazards of hypertension compared to those with normal renal function (creatinine clearance ≥90 ml/min) (HR: 1.70; 95% CI: 1.18–2.46) (Table 5).

**Table 5:** Predictors of incident hypertension in patients on ART after April 2010 when tenofovir was introduced into first-line ART (n = 32,919).

Variable	Number of events/person years	Incidence rate/100 person years (95% CI^¥^)	Crude hazard ratio (95% CI^¥^)	Adjusted hazard ratio (95% CI^¥^)
**Gender**				
Female	1,781/31,985	5.57 (5.31–5.83)	Reference	Reference
Male	1,426/19,936	7.15 (6.79–7.53)	1.28 (1.19–1.37)	1.48 (1.28–1.71)
Age (years) at ART* **initiation**				
18–24	81/2,970	2.73 (2.17–3.39)	0.50 (0.40–0.63)	0.38 (0.22–0.67)
25–29	235/8,477	2.77 (2.43–3.15)	0.54 (0.47–0.62)	0.58 (0.44–0.76)
30–39	1,229/23,659	5.19 (4.91–5.49)	Reference	Reference
40–49	1,054/13,121	8.03 (7.56–8.53)	1.63 (1.50–1.77)	1.55 (1.33–1.80)
≥50	608/3,693	16.46 (15.18–17.83)	2.76 (2.50–3.05)	2.18 (1.78–2.67)
**Body mass index (kg/m**^**2**^) at ART* **initiation**				
<18	205/3,958	5.18 (4.49–5.94)	0.85 (0.73–0.99)	0.88 (0.68–1.12)
18.0–24.9	1,257/23,553	5.34 (5.05–5.64)	Reference	Reference
25.0–29.9	525/7,352	7.14 (6.54–7.78)	1.38 (1.2.4–1.53)	1.65 (1.39–1.95)
30.0–34.9	240/2,602	9.22 (8.09–10.47)	1.77 (1.54–2.04)	2.35 (1.87–2.95)
≥35	103/1,020	10.09 (8.24–12.24)	1.92 (1.57–2.35)	2.15 (1.50–3.07)
Pre-hypertension at ART* **initiation**				
No	2,477/45,945	5.39 (5.18–5.61)	Reference	Reference
Yes	730/5,976	12.22 (11.35–13.14)	2.62 (2.44–2.81)	2.72 (2.38–3.11)
**Non-nucleoside reverse transcriptase inhibitor**				
efavirenz	2,951/48,374	6.10 (5.88–6.32)	Reference	Reference
nevirapine	239/3,249	7.36 (6.45–8.35)	1.20 (1.05–1.37)	1.91 (1.46–2.50)
**Nucleoside reverse transcriptase inhibitor**				
tenofovir	2,262/38,939	5.81 (5.57–6.05)	Reference	Reference
zidovudine	123/1,770	6.95 (5.77–8.29)	1.12 (0.93–1.36)	1.20 (0.75–1.93)
stavudine	822/11,211	7.33 (6.84–7.85)	1.31 (1.21–1.42)	1.33 (1.13–1.58)
CD4 count at ART* **initiation (cells/μl)**				
0–49	555/8,174	6.79 (6.24–7.38)	1.16 (0.97–1.38)	1.23 (0.91–1.67)
50–99	440/6,277	7.01 (6.37–7.70)	1.28 (1.06–1.53)	1.19 (0.87–1.63)
100–199	664/11,359	5.85 (5.41–6.31)	1.19 (1.01–1.42)	1.10 (0.83–1.46)
200–349	795/14,031	5.67 (5.28–6.07)	1.16 (0.97–1.38)	1.09 (0.82–1.45)
350+	157/2,873	5.46 (4.64–6.39)	Reference	Reference
**WHO**^€^ stage at ART* **initiation**				
I/II	1,134/18,724	6.06 (5.71–6.42)	Reference	Reference
III/IV	729/11,365	6.41 (5.96–6.90)	0.96 (0.88–1.06)	1.03 (0.89–1.19)
Hemoglobin at ART* **initiation (g/dL)**				
<10	622/8,861	7.02 (6.48–7.59)	1.01 (0.92–1.10)	1.24 (1.04–1.47)
≥10	2,127/36,047	5.90 (5.65–6.16)	Reference	Reference
**Creatinine Clearance (mL/min/1.73m**^**2**^**)**				
<60	84/512	16.40 (13.08–20.31)	2.11 (1.69–2.64)	1.70 (1.18–2.46)
60–89	234/2,043	11.46 (10.03–13.02)	1.81 (1.58–2.08)	1.34 (1.06–1.70)
≥90	1,844/32,827	5.62 (5.36–5.88)	Reference	Reference

*ART = antiretroviral therapy;

^€^WHO = World Health Organization;

^¥^CI = confidence interval. All predictors are measured at ART initiation. Model adjusted for gender, BMI, age, NRTI, NNRTI, WHO stage, CD4 count at ART initiation, hemoglobin levels at ART initiation, creatinine clearance. Fine and Gray’s competing risk regression used controlling for death as a competing risk for incident hypertension

### Clinical management and outcomes among patients receiving treatment for hypertension

**Fig 2:**
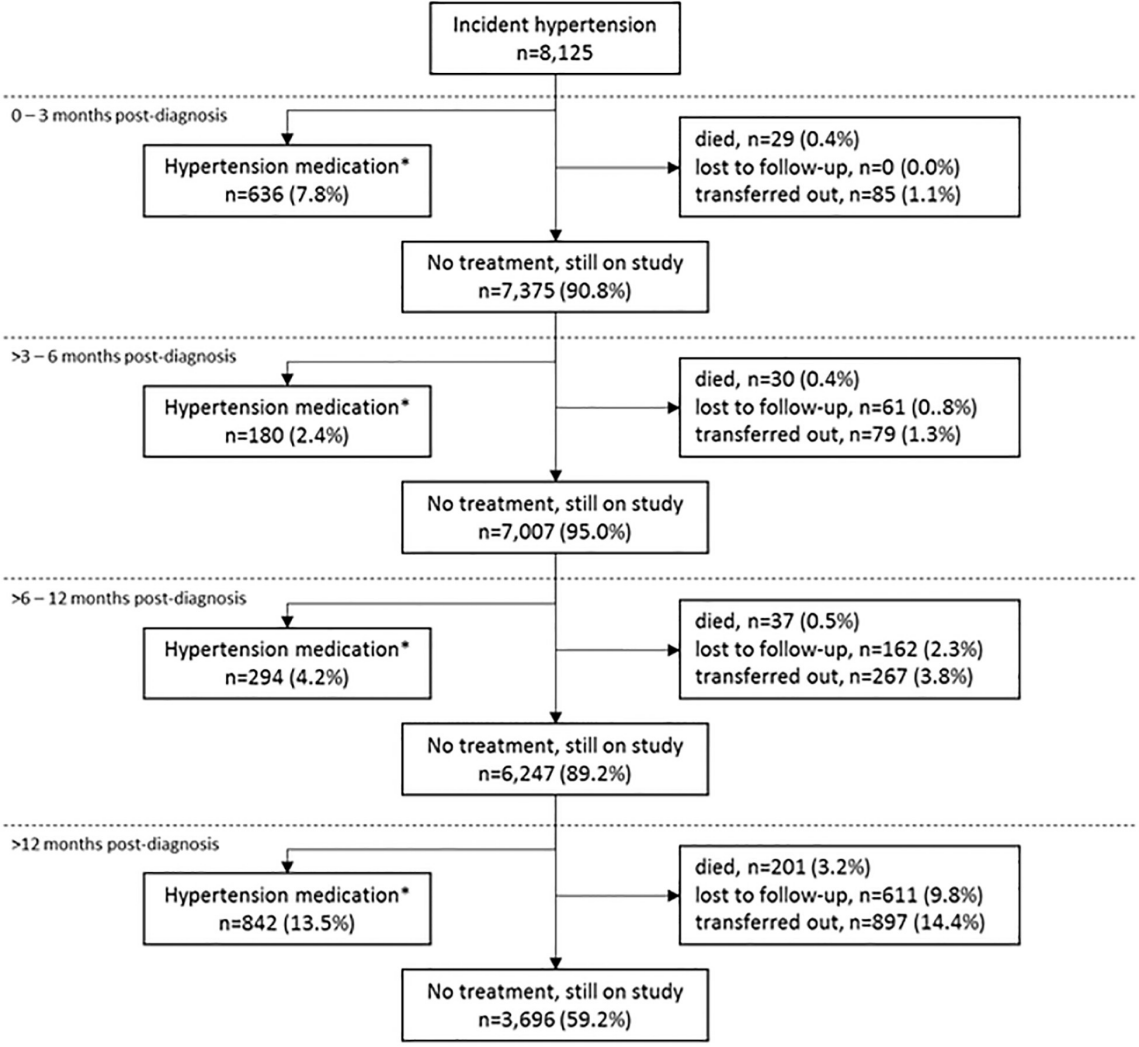
Clinical management of incident hypertension amongst patients on antiretroviral therapy. Hypertension medication includes the following: amlodipine besylate, atenolol, captopril, carvedilol, enalapril maleate, enalaprilat, felodipine, hydralazine hydrochloride, indapamide, lisinopril, metoprolol tartrate, nifedipine, nimodipine, nisoldipine, perindopril erbumine, prazosin hydrochloride, propranolol hydrochloride, verapamil hydrochloride, valsartan, simvastatin.

Of the 8,125 incident hypertension cases, 24.0% (n = 1,952) received medical treatment for elevated blood pressure at the same clinic during the course of follow-up (Fig 2). Of these, 32.6% (n = 636) were treated within 3 months, 9.2% (n = 180) within 3–6 months, 15.0% (n = 294) within 6–12 months, and 43.1% (n = 842) more than 12 months after hypertension diagnosis (Fig 2). Of the 1,952 who received treatment, 4.1% (n = 79) died, 10.6% (n = 207) were lost to follow-up, 16.7% (n = 325) transferred out, and 68.7% (n = 1,341) remained in care at their original clinics after the incident hypertension event while on ART. More than half (58.2%) of the treated patients who had sufficient follow-up blood pressure data (n = 1,546/1,952) went on to have elevated blood pressure at a median of 5 months (IQR 3–9 months) after receiving hypertension medication. A small fraction of these patients (n = 35, 1.8%) developed 37 co-morbidities between them related to hypertension, including 26 (70.3%) cases of hyperlipidemia/ hypercholesterolemia, 7 (18.9%) of hypertensive heart disease, 2 (5.4%) of cardiomyopathy, and 2 (5.4%) of congestive heart failure.

### Clinical management and outcomes among patients not receiving treatment for hypertension

The remaining 6,173 (76.0%) with incident hypertension did not get medical treatment during follow-up. The frequency of mortality (4.8%; n = 298), loss to follow up (13.5%; n = 834) and transfers out of the clinic (22.2%; n = 1,371) were higher than in the population of patients who received treatment for hypertension. 53.9% of these untreated patients with sufficient follow-up blood pressure data (n = 4,849/6,173) still had elevated blood pressure at a median of 6 months (IQR: 5–13) after their initial incident hypertension diagnosis, and 1.6% (n = 96) of them had 98 co-morbidities between them related to hypertension: 93 (94.9%) cases of hyperlipidemia/ hypercholesterolemia, 1 (1.0%) of heart failure, 3 (3.1%) of cardiomyopathy and 1 (1.0%) of cardiac arrest.

As treatment programs for HIV are scaled up globally and deaths from AIDS-defining conditions decline, the management of NCDs, such as hypertension, is emerging as an essential part of HIV care in low- and middle-income countries. Longitudinal data on incident hypertension in HIV patients in sub-Saharan Africa are scarce. With over 75,000 HIV-positive patients on ART over 12 years of follow-up, our cohort is one of the largest to date to assess prevalent and incident hypertension, associated risk factors, treatment, and control of the condition in a middle-income setting. As such, our work adds to the evidence base on hypertension and its risk factors in settings of both high HIV prevalence and the non-HIV risk factors common in middle-income countries.

In the present study, we found a prevalence of hypertension of 22% at ART initiation, consistent with previously published estimates from other sub-Saharan countries [19]. Incidence of hypertension in our cohort was 13% over a median of 22 months of follow up, corresponding to an overall rate of hypertension of 5.4 per 100 person-years. While incidence estimates among HIV-positive populations are scarce, our results are lower than studies conducted in low-income populations in sub-Saharan Africa (12.0 cases per 100 person-years (95% CI: 7.2–15.0) in Tanzania [31]; 11.2 cases per 100 person-years in Uganda (95% CI: 10.2–12.2)) [32] that had similar prevalence. When comparing our rates to the HIV-negative population, our estimates appear to be substantially lower than previous studies out of South Africa have reported. Estimates from the Heart of Soweto Study reported an incidence rate of 24% over 5 years of follow-up [23].

Risk factors for hypertension are well-characterized, and our findings were generally consistent with previous work [32–34]. We found that males, patients age >40 years, those with body mass index ≥25 kg/m^2^, and patients with pre-hypertension at ART initiation were at increased hazards of incident hypertension. Patients with pre-hypertension were twice as likely to experience incident hypertension, underscoring the need to monitor these patients closely. We also found that patients with low CD4 count at ART initiation were at increased hazards of incident hypertension. Previous research showed patients with a nadir CD4 cell count <50 cells/mm^3^ had a 150% (adjusted odds ratio [OR], 2.48; 95%CI: 1.27–4.83) increase in the odds of hypertension compared to those with higher CD4 cell counts [35]. A potential biological mechanism explaining this association is that low CD4 cell count has been associated with chronic immune activation and persistent microbial translocation [35–39]. Ingjerd et al. demonstrated a strong association between microbial translocation, measured by increased levels of lipopolysaccharide before initiation of ART, and subsequent sustained hypertension [40]. However, this association was inverted when assessing hypertension at ART initiation, where we show that more advanced disease (WHO stage III/IV, low CD4 and low Hb) was protective against prevalent hypertension. Weight loss and lower levels of triglycerides, most frequently found in patients with CD4 lymphocyte counts of less than 200 cells/mm^3^ [41,42], could be reflected in the lower prevalence of incident hypertension in patients on ART. Our linear regression models, which used standardized data to assess predictors of systolic and diastolic blood pressure, showed minimal effect of any risk factors.

Consistent with previous studies [43,44], we observed a 30% increased hazards of hypertension among patients on nevirapine compared to efavirenzand a 40% increased hazards of hypertension in patients on stavudine or zidovudine vs tenofovir. Although we were unable to assess the association of incident nephrotoxicity on incident hypertension, we did assess the association of hypertension with renal function at ART initiation and found that patients with moderate/severe renal insufficiency (HIV-associated, not ART related, nephropathy) at treatment initiation had a 55% increased hazard of incident hypertension compared to those with normal renal function. As two potential pathways that lead to increased risk of hypertension risk amongst HIV patients, researchers speculate that ART effects body mass index or immune reconstitution [42,45]. Studies have also shown that exposure to tenofovir has been associated with accelerated eGFR decrease in the first 6 to 12 months after ART initiation among African HIV patients with no or mild renal dysfunction at initiation of ART [46]. The possible impact of secondary nephrotoxicity on hypertension rates deserves consideration in view of the large proportion of patients receiving tenofovir-based ART since it became standard first-line ART in 2010 in many low- and middle-income countries [47].

In low- and middle-income countries, many people do not seek treatment for hypertension because it is prohibitively expensive due to the cost of the drugs. Close to 25% of patients in our cohort who had incident hypertension during follow up observation received medication for the condition. Of these, close to 60% had high blood pressure five months after treatment, suggesting that either the medication was not effective or was not adhered to appropriately. More than half of the remaining 75% of patients with incident hypertension, and no documentation of treatment for the condition, went on to have normal blood pressure measurements within six months of their diagnosis. The goal of the national guidelines for monitoring and treatment of hypertension is to decrease blood pressure to <140/90 mmHg in patients being treated with antihypertensive medication regardless of cardiovascular risk factors and underlying co-morbidities [48]. Further research is needed to closely examine the monitoring and treatment of the condition in patients receiving long-term ART.

Our findings should be considered alongside the study’s limitations. First, because our study reports data from large, public sector government HIV clinics, our results may not be generalizable to the overall population. Second, lack of documentation of blood pressure measurements, clinical hypertension diagnosis, and/or prescribed antihypertensive medication could cause us to underestimate the proportion of patients prevalent and incident hypertension in the sample. This missingness could be the result of poor data capturing at the clinic level or due to the fact that monitoring and treatment of NCDs occurs separately from HIV care so a known hypertensive patient on treatment may not be reflected in the HIV data. Third, this lack of accurate information on prescribed hypertensive medication could explain why 58.2% of patients that received treatment for incident hypertension and went on to have elevated blood pressure at a median of 5 months after receiving hypertension medication, while 53.2% of those that did not receive treatment had elevated blood pressure measurement in a median of 6 months after being diagnosed hypertensive. Fourth, patients that were initiated onto the older generation of antiretroviral drugs (nevirapine, stavudine or zidovudine) could have had contraindications to efavirenz or tenofovir, placing them at higher hazards of hypertension. Since information on conditions that would indicate contraindication to the newer antiretrovirals was not available, we were unable to control for this and may be overestimating the hazards of hypertension in patients on those antiretrovirals. Fifth, we show that more advanced disease (WHO stage III/IV, low CD4, and low Hb) is protective against prevalent hypertension, while low BMI (<18 kg/m^2^) is protective against incident hypertension. This might be related to BMI, as when adjusting for BMI our estimates are slightly attenuated, but still remain significant. It may be that BMI is not as accurate a marker of body fat as waist measurement would be [49] and adjusting for waist could nullify the effect association of makers of advanced disease. Alternatively, these indicators of poorer health status by WHO classification could be associated with malnutrition, in which low blood pressure is common, implying that our cohort differs from healthy HIV-negative cohorts in that it contains malnourished, HIV-infected patients, both at the onset of ART and over a life time of treatment. Six, as our estimates of prevalent hypertension are among HIV-positive patients that survive to initiate ART, survivor bias result in an underestimate of prevalence, biasing our results downwards. Finally, as we do not have access to HIV-negative population for comparison, we are unable to assess any association between HIV status and hypertension. However, the 2016 South African Demographic and Health Survey reported estimated a hypertension prevalence of over 40% among black South Africans older than 15 years of age [10], roughly 20% higher than the estimated 22% we reported in this study and consistent with previous research from sub-Saharan Africa [9,23].

## Conclusion

Over 20% of patients in our cohort had hypertension at ART initiation, and 13% of those with normal blood pressure at ART initiation developed hypertension while on ART. Older patients, males, those on nevirapine, zidovudine or stavudine, and those who are overweight/obese should be targeted for frequent blood pressure monitoring and the identification of other cardiovascular risk factors to encourage lifestyle modifications. Additionally, these at-risk populations should be offered pharmaceutical therapy to help prevent myocardial infarction, heart failure, stroke, and kidney disease. Further research is needed to determine the level of access and adherence to pharmaceutical treatment for hypertension in this population. Additionally, an HIV-negative comparison population is needed to assess the association of the HIV virus itself with hypertension.
